# When Does Return of Voluntary Finger Extension Occur Post-Stroke? A Prospective Cohort Study

**DOI:** 10.1371/journal.pone.0160528

**Published:** 2016-08-05

**Authors:** Caroline Winters, Gert Kwakkel, Rinske Nijland, Erwin van Wegen, EXPLICIT-stroke consortium

**Affiliations:** 1 Department of Rehabilitation Medicine, VU University Medical Center, MOVE Research Institute, Amsterdam, The Netherlands; 2 Neuroscience Campus Amsterdam, Vrije Universiteit, Amsterdam, The Netherlands; 3 Amsterdam Rehabilitation Research Center, Reade, Amsterdam, The Netherlands; 4 Department of Physical Therapy and Human Movement Sciences, Northwestern University, Evanston, IL, United States of America; "INSERM", FRANCE

## Abstract

**Objectives:**

Patients without voluntary finger extension early post-stroke are suggested to have a poor prognosis for regaining upper limb capacity at 6 months. Despite this poor prognosis, a number of patients do regain upper limb capacity. We aimed to determine the time window for return of voluntary finger extension during motor recovery and identify clinical characteristics of patients who, despite an initially poor prognosis, show upper limb capacity at 6 months post-stroke.

**Methods:**

Survival analysis was used to assess the time window for return of voluntary finger extension (Fugl-Meyer Assessment hand sub item finger extension≥1). A cut-off of ≥10 points on the Action Research Arm Test was used to define return of some upper limb capacity (i.e. ability to pick up a small object). Probabilities for regaining upper limb capacity at 6 months post-stroke were determined with multivariable logistic regression analysis using patient characteristics.

**Results:**

45 of the 100 patients without voluntary finger extension at 8 ± 4 days post-stroke achieved an Action Research Arm Test score of ≥10 points at 6 months. The median time for regaining voluntary finger extension for these recoverers was 4 weeks (lower and upper percentile respectively 2 and 8 weeks). The median time to return of VFE was not reached for the whole group (N = 100). Patients who had moderate to good lower limb function (Motricity Index leg≥35 points), no visuospatial neglect (single-letter cancellation test asymmetry between the contralesional and ipsilesional sides of <2 omissions) and sufficient somatosensory function (Erasmus MC modified Nottingham Sensory Assessment≥33 points) had a 0.94 probability of regaining upper limb capacity at 6 months post-stroke.

**Conclusions:**

We recommend weekly monitoring of voluntary finger extension within the first 4 weeks post-stroke and preferably up to 8 weeks. Patients with paresis mainly restricted to the upper limb, no visuospatial neglect and sufficient somatosensory function are likely to show at least some return of upper limb capacity at 6 months post-stroke.

## Introduction

Voluntary finger extension (VFE) is an important early predictor of recovery of upper limb capacity at 6 months post-stroke[1;2]. Patients without VFE within the first days post-stroke have been suggested to have a poor prognosis for regaining some upper limb capacity at 6 months[1–3]. Absence of VFE reflects the loss of functional corticospinal tract integrity, acknowledging that the hand muscles are almost solely innervated by contralateral corticospinal pathways[4]. Indirect bilateral innervation of the hand muscles by the reticulospinal tract may also contribute to hand motor control after stroke[5]. However, it remains unclear if the reticulospinal system can influence the digital extensor muscles of the paretic hand[6].

Despite an initially poor prognosis, some patients without VFE within the first days after stroke do regain upper limb capacity at 6 months[2]. In view of the lack of evidence-based therapies for patients without VFE[7;8], this return of VFE seems most likely to be driven by spontaneous neurobiological processes such as alleviation of diaschisis[9]. Unfortunately, the clinical characteristics as well as the optimal time window for recovery of VFE are unknown, due to lack of prospective cohort studies in which patients are assessed serially at fixed times post-stroke[10;11]. More knowledge regarding this time window is important for future prognostic algorithm development. Up till now, the most optimal timing and added value of neurophysiological and neuroimaging measurements with respect to clinical measurements like VFE are unclear.

The aims of the present study were therefore (1) to determine the clinical time window for return of VFE in ischemic stroke patients without VFE in the first days post-stroke, and (2) to identify clinical characteristics for the return of some upper limb capacity in these patients within the first 6 months after stroke. We hypothesized that return of VFE would occur within the purported time window of spontaneous neurobiological recovery between 0 and 10 weeks after stroke onset[10;12]. We also hypothesized that patients with lesions affecting upper limb function who exhibit no other neurological impairments such as visuospatial neglect and somatosensory dysfunction would have a high probability of regaining some upper limb capacity at 6 months[13–15].

## Materials and Methods

### Recruitment

Data from the EXPLICIT-stroke program was used[16;17]. EXPLICIT-stroke was a Dutch translational research program including two multi-center single blinded randomized trials and an intensive repeated measurements design up to 6 months post-stroke. Between October 2008 and November 2013 a total number of 159 patients were included. For the present study only patients in the EMG-triggered neuromuscular stimulation (EMG-NMS) trial were selected (N = 101). Patients were recruited within the first 2 weeks post-stroke and allocated to control treatment (usual care) or experimental treatment focused on regaining VFE (EMG-NMS). Details of the EMG-NMS treatment protocol have been described elsewhere.[16;17] EXPLICIT-stroke was approved by the ethics committee of all participating centers (Leiden University Medical Center: No. P08.035; Dutch Central Committee on Research Involving Human Subjects [CCMO]: No. NL21396.058.08) and was registered at the Netherlands Trial Register (TC1424).

### Subjects

Patients were included when they met the following criteria upon screening: (1) first-ever ischemic middle cerebral artery stroke verified by CT and/or MRI scan; (2) upper limb paresis according to National Institutes of Health Stroke Scale item 5 (score >0); (3) no VFE at baseline according to Fugl-Meyer Assessment hand sub question FE (score = 0); (4) mini mental state examination ≥23; (5) 18–80 years of age; (6) no upper limb musculoskeletal impairments; (7) no botulinum toxin treatment, as this may distort the measurement of upper limb capacity; (8) able to sit independently for 30 seconds, i.e. sufficient sitting balance to facilitate clinical measurements; (9) written informed consent.

### Serial Assessments

Eight repeated assessments were performed using the Fugl-Meyer Assessment hand sub question FE to monitor return of VFE (0 = no movement, 1 = partial movement, 2 = full movement)[18]. Assessments were performed weekly in the first 5 weeks post-stroke and at 8, 12 and 26 weeks.

### Dependent Variable

The Action Research Arm Test (ARAT) served as outcome measure at 6 months post-stroke. The ARAT is a capacity test in the activities domain of the International Classification of Function, Disability and Health framework[19] and includes 19 tasks divided into 4 subdomains: grasp, grip, pinch and gross movement (maximum = 57 points). The ARAT has a maximal score of 57 points and good clinimetric properties[20;21].

### Independent Variables

The following independent baseline variables were identified based on previous literature[22]: (1) Sex; (2) Age; (3) Hemisphere of stroke; (4) Bamford classification[23]; (5) Time between stroke and baseline assessment; (6–7) Hemianopia and facial palsy (National Institutes of Health Stroke Scale)[24]; (8) Visuospatial neglect (Letter Cancellation Test)[25], the presence of visuospatial neglect being defined as an asymmetry between the contralesional and ipsilesional body sides of ≥2 omissions[14]; (9) Somatosensory function of the upper limb (touch, sharp-blunt discrimination and proprioception of the Erasmus MC modified Nottingham Sensory Assessment)[26]; and (10–12) Range of motion and strength of the elbow, shoulder, and lower limb (Motricity Index)[27]. In addition, we added the EXPLICIT-stroke randomization group as independent variable: experimental (EMG-NMS) versus control group.

### Data Analysis

We used survival analysis on the repeated assessments to determine the time until patients regained VFE for the first time (i.e. partial or full movement according to the Fugl-Meyer Assessment hand sub item FE). For those patients with missing data we took the first assessment where VFE was measured as ‘time to return of VFE’. Possibly, these patients could have regained VFE earlier in time. Recovery was defined as an ARAT score of ≥10 points, representing at least some upper limb capacity, i.e. patients should at least be able to pick up a small object against gravity (dichotomization: 0 = ARAT<10 and 1 = ARAT≥10)[2;28]. A Kaplan-Meier [29] cumulative ‘event’ curve was constructed for the whole group and for the patients with 10 or more points on the ARAT at 6 months post-stroke.

For our second objective, statistical analyses were performed on subjects with complete baseline and 6-month assessments. Dichotomization of independent variables was preferably based on clinical reasoning or previous literature[2]. Otherwise, we used the Youden index to determine the cut-off point with the highest sensitivity and specificity[30]. Bivariable logistic regression analysis was used to preselect independent variables when the Wald statistic was *p*<0.05. Subsequently, collinearity diagnostics between preselected variables was applied using two-way contingency tables. If the Phi correlation coefficient between two variables was ≥0.8, the variable with the lowest Wald statistic was excluded from further analysis. Thereafter, we used a backward stepwise multivariable logistic regression analysis on the selected variables to form the prediction model (entry criteria = *p*<0.05; removal criteria = *p*≥0.10). In view of the large number of preselected variables relative to the number of patients included in the study, we applied a forward stepwise approach to test model stability. The Hosmer-Lemeshow test and the *c-*statistic (i.e. area under the receiver-operating characteristic curve) were used to quantify the goodness-of-fit of the logistic regression model, and two-way contingency tables to calculate sensitivity, specificity, positive predictive value, and negative predictive value, including the corresponding 95%CI. Statistical analyses were two-tailed with an alpha of 0.05 (SPSS version 20).

## Results

One hundred one first-ever ischemic stroke patients were recruited for the EXPLICIT-stroke EMG-NMS trial. In the present study, 1 patient was excluded for further analyses due to presence of some VFE. Patient characteristics of the 100 patients eligible for further analysis are shown in Table 1. At 6 months post-stroke, 45 of the 100 patients achieved an ARAT score of ≥10 points. This group of patients had a baseline ARAT score ranging from 0 points to 7 points, with 30 patients scoring 0 points, 11 patients scoring between 1 and 5 points, and 4 patients scoring 6 or 7 points. At 6 months post-stroke, this group had a median ARAT score of 34 points (interquartile range [IQR] = 19.50–45.00; range = 10–57); 4 patients scored 10 points, and 5 of them attained the maximum score of 57 points. In comparison, the median 6-month ARAT score for the other group was 0 (IQR = 0–3; range = 0–4).

**Table 1:** Patient characteristics.

Baseline	N = 100
Sex: female/male, %male	36/64, 64%
Age, years^†^	58.67 (11.72)
Hemisphere of stroke: left/right, %right	31/69, 69%
Bamford classification: LACI/PACI/TACI	55/39/6
Global disability (NIHSS, range:0–40) (N = 99)^*^	9 (7–11)
Hemianopia (NIHSS): no/yes, %yes	92/8, 8%
Facial palsy (NIHSS): no/yes, %yes	15/85, 85%
Extinction and inattention (NIHSS): no/yes, %yes	68/32, 32%
Sensation (NIHSS): no/yes, %yes	52/48, 48%
Visuospatial neglect (LCT): no/yes, %yes (N = 94)	36/58, 58%
Somatosensory function (EmNSA): good/poor, %poor (N = 99)	57/42, 42%
Upper limb function (MI, range:0–100)^*^	0 (0–23)
Upper limb function (FMA, range:0–66)^*^	5 (4–8)
Lower limb function (MI, range:0–100) (N = 99)^*^	32 (9–47)
Upper limb capacity (ARAT, range:0–57)^*^	0 (0–0)
Time between stroke and baseline assessment, days^†^	8.26 (4.10)
**6 months post-stroke**	
Upper limb capacity (ARAT, range:0–57, N = 97)^*^	4 (0–30.5)
Time between stroke and 6-month assessment, days^†^	189.90 (14.10)

Data from all 100 subjects, unless otherwise indicated. LACI = Lacunar Anterior Cerebral Infarction; PACI = Partial Anterior Cerebral Infarction; TACI = Total Anterior Cerebral Infarction; NIHSS = National Institutes of Health Stroke Scale; LCT = Letter Cancelation Test; EmNSA = Erasmus MC modified Nottingham Sensory Assessment; MI = Motricity Index; FMA = Fugl-Meyer Assessment. Data presents number of patients (N)

^*^median (IQR)

^†^mean (SD).

**Fig 1:**
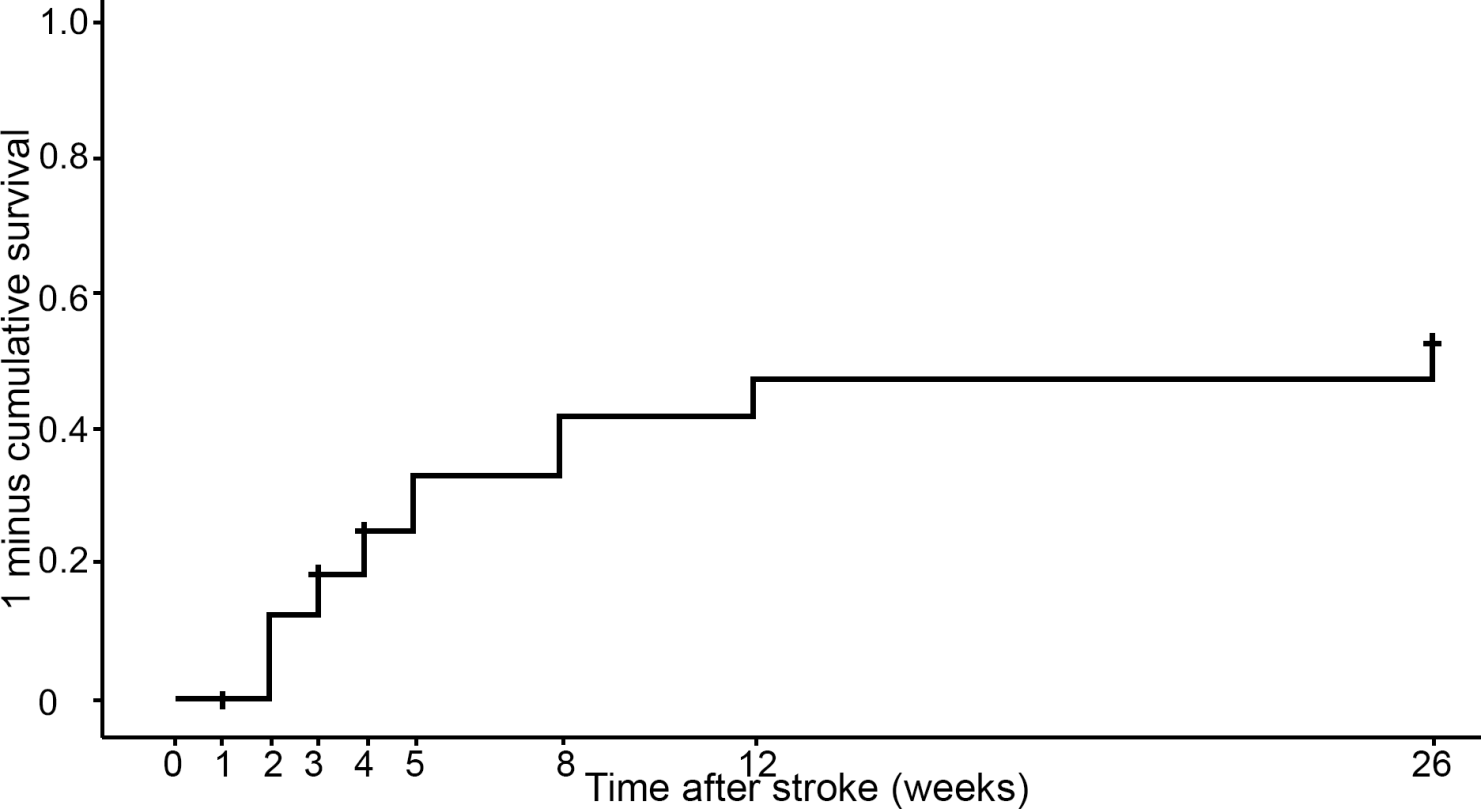
Kaplan-Meier cumulative ‘event’ curve for recovery of VFE (N = 100). The numbers represent the number of patients with VFE at each time point (Fugl-Meyer Assessment hand sub item FE≥1). Three patients were lost to follow-up.

**Fig 2:**
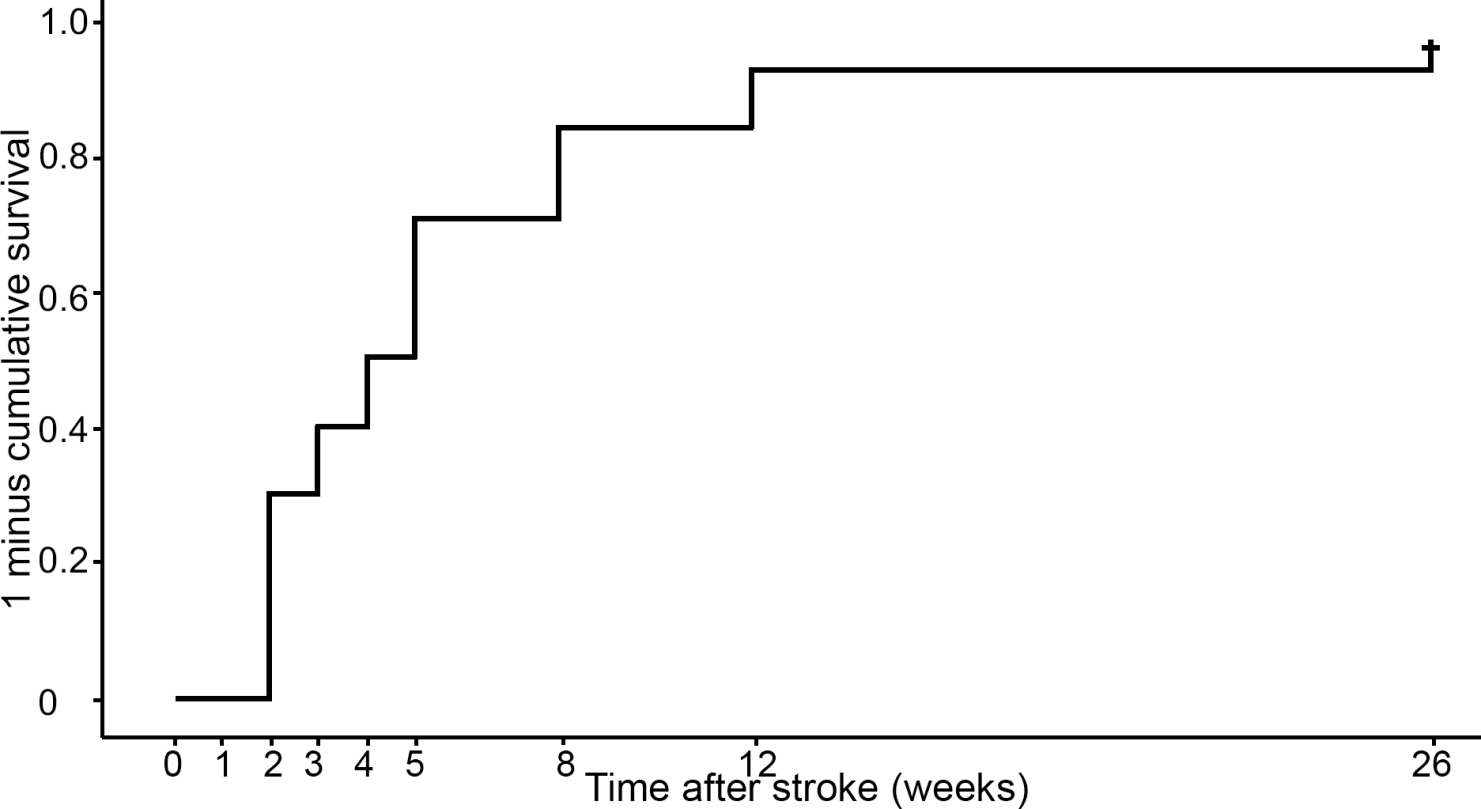
Kaplan-Meier cumulative ‘event’ curve for recovery of VFE in the group of patients who regain some upper limb capacity at 6 months post-stroke (N = 45). The numbers represent the number of patients with VFE at each time point (Fugl-Meyer Assessment hand sub item FE≥1).

Approximately 7% of the repeated assessments of VFE were missing. The Kaplan-Meier curve for the whole group of 100 patients is shown in Fig 1. Three patients were lost to follow-up due to death, recurrent stroke and withdrawal. The median time to return of VFE was not reached within the first 26 weeks post-stroke. Visually, we do see that return of VFE occurs primarily within the first 8 weeks. Note that the cumulative ‘event’ curve is 1 minus survival, and resembles the probability of an event. In other words, a high and/or rapidly rising curve is a favorable outcome (i.e. return of FE). An additional analysis was performed on the group of patients who did regain some upper limb capacity (i.e. ARAT ≥ 10 points) at 6 months post-stroke (N = 45, Fig 2). The Kaplan-Meier curve in Fig 2 shows an initial sharp rise and reaches a median time to return of VFE at 4 weeks post-stroke (SE = 0.52; 95%CI = 2.99–5.01; Fig 2). The lower (25^th^) and upper (75^th^) percentiles were respectively 2 weeks and 8 weeks post-stroke. Twenty-three patients (51%) of the recoverers had regained VFE at 4 weeks after stroke, and at 8 weeks this number had increased to 38 patients (84%).

Logistic regression analyses were only performed on patients with complete baseline and 6-month assessments (N = 91); we excluded 3 drop-outs as described before and another 6 patients due to missing baseline data. Bivariable logistic regression analysis showed that 7 of the 13 baseline variables were significantly related to recovery of upper limb capacity (ARAT≥10 points) at 6 months post-stroke (*p*<0.05, Table 2). Collinearity diagnostics between independent variables showed no correlation coefficients ≥0.8. The subsequent backward multivariable logistic regression analysis, performed on the 7 preselected baseline variables, yielded a prediction model for return of upper limb capacity despite initial lack of VFE consisting of 3 variables: (1) moderate to good lower limb motor function, i.e. Motricity Index-leg≥35 points; (2) absence of visuospatial neglect, i.e. a letter cancellation test asymmetry between the contralesional and ipsilesional sides of <2 omissions; and (3) sufficient somatosensory function, i.e. Erasmus MC modified Nottingham Sensory Assessment≥33 points. Forward stepwise analysis confirmed these results. Table 3 displays all predicted probabilities of the model, ranging from 0.04–0.94, with a Hosmer-Lemeshow chi-square of 3.41(*p* = 0.637) and a c-statistic of 0.89. Two-way contingency tables showed a sensitivity of 0.88 (95%CI = 0.74–0.96), specificity of 0.73 (95%CI = 0.60–0.85), positive predictive value of 0.74 (95%CI = 0.60–0.85) and negative predictive value of 0.88 (95%CI = 0.74–0.96) for the final model.

**Table 2:** Candidate baseline determinants associated with regaining some upper limb capacity at 6 months post-stroke.

	N = 91		
	Odds Ratio	95%CI	*p*
Sex (0 = female; 1 = male)^†^	0.79	0.33–1.91	0.606
Age (years)^‡^	1.00	0.97–1.04	0.950
Hemisphere of stroke (0 = right; 1 = left)^†^	2.40	0.94–6.10	0.066
Type of stroke (Bamford classification: 0 = PACI/TACI;1 = LACI)^†^	3.33	1.39–8.01	0.007*
Time between stroke and baseline (days)^‡^	1.07	0.96–1.18	0.222
Hemianopia (NIHSS item-3:0 = yes; 1 = no)^†^	0.85	0.16–4.44	0.845
Facial palsy (NIHSS item-4:0 = yes; 1 = no)^†^	3.99	1.16–13.69	0.028*
Visuospatial neglect (LCT asymmetry: 0≥2; 1<2)^§^	6.54	2.53–16.90	<0.001*
Somatosensory function (EmNSA:0<33; 1≥33)^d^	4.06	1.69–9.78	0.002*
Shoulder abduction (MI: 0<9; 1≥9)^†^	3.68	1.54–8.79	0.003*
Elbow flexion (MI: 0<9; 1≥9)^†^	8.00	2.65–24.17	<0.001*
Lower limb function (MI-leg: 0<35; 1≥35)^||^	12.67	4.68–34.32	<0.001*
Randomization (0 = control group; 1 = experimental group) ^†^	0.80	0.35–1.84	0.605

PACI = Partial Anterior Cerebral Infarction; TACI = Total Anterior Cerebral Infarction; LACI = Lacunar Anterior Cerebral Infarction; NIHSS = National Institutes of Health Stroke Scale; LCT = Letter Cancellation Test; EmNSA = Erasmus modified Nottingham Sensory Assessment; MI = Motricity Index.

*Wald statistic = *p*<0.05

^†^Based on clinical grounds

^‡^Not dichotomized

^§^Based on previous literature(14)

^||^Based on area under the receiver-operating characteristic curve.

**Table 3:** Probabilities of regaining some upper limb capacity at 6 months post-stroke in patients who initially did not show finger extension.

LL	VSN	SSF	True Negatives (N)	False Negatives (N)	False Positives (N)	True Positives (N)	Predicted probability (0–1)
Good	No	Good	36	5	13	37	0.94
Good	No	Poor					0.81
Good	Yes	Good					0.72
Poor	No	Good					0.51
Good	Yes	Poor					0.39
Poor	No	Poor					0.21
Poor	Yes	Good					0.13
Poor	Yes	Poor					0.04

Model: P(upper limb capacity) = 1/1+e^-(-3.24+2.80xLL+1.91xVSN+1.36xSSF)^. LL = lower limb function (motricity index leg); VSN (letter cancellation test asymmetry); SSF = somatosensory function (Erasmus MC modified Nottingham Sensory Assessment).

## Discussion

To our knowledge, this is the first study to prospectively investigate the clinical time window for spontaneous return of VFE in ischemic stroke patients who showed no VFE within the first days post-stroke. The present study shows that if patients do regain VFE, this will most likely occur within the first 8 weeks after stroke onset. However, more than half of the patients who regained some upper limb capacity at 6 months post-stroke already showed this return of VFE within the first 4 weeks post-stroke. In view of the absence of evidence-based therapies for this specific group of patients[7;8], as well as our neutral trial showing no effects of early-start EMG-NMS intervention[17], we believe this return of VFE is most likely driven by spontaneous processes of neurological recovery.

Acknowledging that most evidence-based therapies for the upper limb depend on selecting patients for their ability to voluntarily extend their fingers, we recommend that clinicians monitor these stroke patients for return of VFE weekly, at least up to 8 weeks post-stroke[31]. This recommendation is in line with the recently developed *Post Stroke Arm Algorithm* application (“app”) which offers clinicians an online tool to select the most appropriate evidence-based upper limb therapy for an individual patient, with an emphasis on the regular monitoring of VFE[31]. If VFE returns within the time window for neurological recovery, the prognosis changes in favor of regaining upper limb capacity, and therapy may focus on improving motor function through high-intensity repetitive exercise to prevent learned non-use of the paretic arm, for example with (modified) constraint-induced movement therapy[32].

Importantly, we do not claim that return of VFE always occurs within the first 8 weeks, as there may be exceptions due to neglect[33], acknowledging the suppressive effects of neglect on motor recovery[14;15]. However, the present study suggests that the likelihood of recovery after this time period is small. Future studies should further investigate those cases that constitute an exception to the rule that is focus on patients who show return of VFE beyond the first 8 weeks post-stroke.

Concerning our second objective, we found that patients with no initial VFE who showed moderate to good lower limb motor function, no visuospatial neglect, and sufficient somatosensory function have a high probability (0.94) of regaining at least some upper limb capacity at 6 months post-stroke (i.e. ability to pick up a small object). The model presented here may be seen as an important contribution to the prediction of upper limb capacity in patients with an initial poor prognosis early post-stroke.

Severity of lower limb paresis was previously suggested to be an important predictor of regaining upper limb function[13;28;34] and may reflect the extent of the lesion and as such the severity of initial neurological damage. The substantial negative impact of visuospatial neglect on upper extremity motor recovery which was found in other studies[15;35;36] may reflect the suppressive effects of the dysfunctional site of injury on anatomically and functionally related brain areas at remote distance from the location of the infarct[37]. Our model also suggests that somatosensory dysfunction is a crucial factor that prevents upper limb recovery [14;37]. Repeated, systematic monitoring of neurological impairments within the first 8 weeks after stroke onset may give more insight in the underlying logistic pattern of spontaneous neurobiological recovery.

### Study Limitations & Future Research

It should be noted that half of the patients in the current study received 3-weeks of additional therapy with EMG-NMS focused at regaining VFE, in comparison to the other group that solely received usual care according to evidence-based guidelines[7]. We postulate that this did not affect our results, as the EXPLICIT-stroke trial did not show any significant interaction effects of time with EMG-NMS on the likelihood of return of upper limb capacity within this time window[17]. High quality randomized clinical trials are needed to determine if the likelihood and timing of this return of VFE can be improved and accelerated by innovative therapies that may enhance neuronal activity and restore activity homeostasis, such as transcranial direct-current stimulation, repetitive transcranial magnetic stimulation or pharmaceuticals combined with exercise therapy.

Second, the present study shows that in particular patients with a paresis of the arm without somatosensory dysfunction and neglect are more likely to have some return of upper limb capacity. The sensitivity of the current model was quite good (0.88, 95%CI = 0.74–0.96), however, the specificity was somewhat lower (0.73, 95%CI = 0.60–0.85). This misclassification of patients as recoverers was also observed in previous studies using transcranial magnetic stimulation (TMS)[38;39]. Moreover, the presence of a motor evoked potential does not necessarily mean that patients will show recovery of hand function[38;39]. Preliminary results from the PREP-algorithm, which sequentially combines clinical measurements of shoulder abduction and finger extension (SAFE) with TMS and diffusion tensor imaging (DTI), also showed a specificity and sensitivity of respectively 0.88 and 0.73[40]. However, these values were for the ‘complete recovery’ subcategory (i.e. ARAT score of 50 points or higher). Sensitivity and specificity values for the other categories (i.e. notable, limited and no recovery) were not provided. We therefore cannot directly compare the preliminary results from the PREP-algorithm with our results as the cut-off values are different. The current results emphasize the importance of repeated clinical assessment of VFE in the first weeks post-stroke for clinical decision making. Within the PREP-algorithm, SAFE was however not reassessed when TMS and DTI were performed at about 2 weeks post-stroke[40]. Determination of confidence intervals reflecting precision of this algorithm, as well as cross-validation are needed to underpin the robustness of propagated models. Future cohort studies are needed to refine our clinical prediction model by exploring the added value of neuroimaging, such as stroke volume or localization (e.g. cortical or subcortical)[41;42] and DTI[43], TMS[39] and other biomarkers associated with the recovery of upper limb capacity after stroke[11].

Third, the sample size of the present study was relatively small in relation to the number of variables. However, model stability and robustness was confirmed with an additional forward stepwise logistic regression analysis.

Fourth, although models with dichotomized outcomes are often used within clinical practice, they do limit the understanding of individual recovery profiles. Moreover, with the current model we cannot differentiate between patients who regain some upper limb capacity (ARAT = 10 points) and those who regain full upper limb capacity (ARAT = 57 points). The cut-off value of 10 or more points on the ARAT was used in previous literature as it represents the recovery of some dexterity[2;28]. Future studies are needed to identify subgroups of patients achieving notable or limited dexterity at 3 and 6 months post-stroke. To achieve this goal, larger prospective cohorts are needed to test the precision and with that the robustness of current models for identifying these subgroups with limited and notable recovery.

Finally, the present study was restricted to patients with a first-ever ischemic middle cerebral artery stroke and did not include cross-validation of the logistic regression model.
